# Chronic eosinophilic pneumonia presenting with ipsilateral pleural effusion: a case report

**DOI:** 10.1186/s13256-016-1005-5

**Published:** 2016-08-12

**Authors:** Narin Sriratanaviriyakul, Hanh H. La, Timothy E. Albertson

**Affiliations:** 1 University of California, Davis, USA; 2 Department of Internal Medicine, Division of Pulmonary, Critical Care and Sleep Medicine, 4150 V Street, Suite 3100, Sacramento, CA 95817 USA; 3 VA Northern California Health Care System, 10535 Hospital Way, Mather, CA 95655 USA; 4 The Queen’s Medical Center, Department of Internal Medicine, 1301 Punchbowl Street, Honolulu, HI 96813 USA; 5 Division of Hematology and Oncology, 4501 X Street, Sacramento, CA 95817 USA

**Keywords:** Chronic eosinophilic pneumonia, Pleural effusion

## Abstract

**Background:**

Chronic eosinophilic pneumonia is a rare idiopathic interstitial lung disease. The nearly pathognomonic radiographic finding is the peripheral distribution of alveolar opacities. Pleural effusions are rarely seen. We report a case of chronic eosinophilic pneumonia with transudative eosinophilic pleural effusion.

**Case presentation:**

A 57-year-old Hispanic woman, a nonsmoker with a history of controlled asthma, presented to the hospital with unresolving pneumonia despite three rounds of antibiotics over a 2-month period. She was later diagnosed with chronic eosinophilic pneumonia based on the presence of peripheral blood eosinophilia, the peripheral distribution of alveolar infiltrates on chest radiograph, and a lung parenchymal biopsy with infiltrates of eosinophils. Upon presentation, our patient had a right-sided moderate-sized pleural effusion. The pleural fluid profile was consistent with a transudative effusion with eosinophil predominance. Our patient responded promptly to oral corticosteroid treatment in a few days. The pulmonary infiltrates and pleural effusion subsided on a 1-month follow-up chest radiograph after starting corticosteroid treatment.

**Conclusions:**

We report the first case of chronic eosinophilic pneumonia presenting with pneumonia with ipsilateral transudative eosinophilic pleural effusion. Like other cases of chronic eosinophilic pneumonia, early recognition and diagnosis is essential and prompt treatment with corticosteroids is the mainstay of therapy. Pleural effusion resolved without the further need for therapeutic thoracentesis.

## Background

Chronic eosinophilic pneumonia (CEP) is a rare disorder, accounting for approximately 2.5 % of interstitial lung disease [1]. It is idiopathic and can occur in any age group but is rarely seen in childhood [2]. Up to half of CEP cases have a history of asthma preceding CEP. Clinical manifestations are nonspecific with subacute to chronic respiratory symptoms being the common presentation. The presence of peripheral blood eosinophilia and characteristic radiographic findings in a patient with pneumonia that fails to resolve with antibiotic treatment should raise the suspicion of CEP and other pulmonary infiltrates with eosinophilia syndromes such as CEP, allergic bronchopulmonary aspergillosis, fungal and parasitic infections, eosinophilic granulomatosis with polyangiitis, hypereosinophilic syndrome.

CEP has a distinctive radiographic feature, which includes peripheral parenchymal opacities involving the upper lobes [3]. The presence of pleural effusion is a very rare finding. Here we report a rare case of CEP presenting with ipsilateral transudative eosinophilic pleural effusion.

## Case presentation

A 57-year-old Hispanic woman, a never smoker with a 20-year past medical history of well-controlled asthma, presented with fever, productive cough, and fatigue for 3 months. Her symptoms did not improve with three courses of antibiotics that included levofloxacin and doxycycline over these 3 months. She also reported symptoms of night sweats and a 10-lb weight loss during this period. She denied sick contacts and any significant environmental exposure history. She had a full-time job as an office worker. Two weeks prior to the presentation her activities of daily living (ADL) were limited to short-distance ambulation within her house. On the day of admission, our patient was not able to get out of bed due to shortness of breath. Her vital signs included a temperature of 38.9 °C, a blood pressure of 99/69 mmHg, a heart rate of 91 beats/min, respiratory rate of 18 breaths/min, and an oxygen saturation of 94 % on 3 L nasal cannula. A physical examination revealed an ill-appearing woman. A pulmonary examination was notable for increased breath sounds and crackles at right middle chest and decreased breath sounds at the right lower chest.

**Fig. 1:**
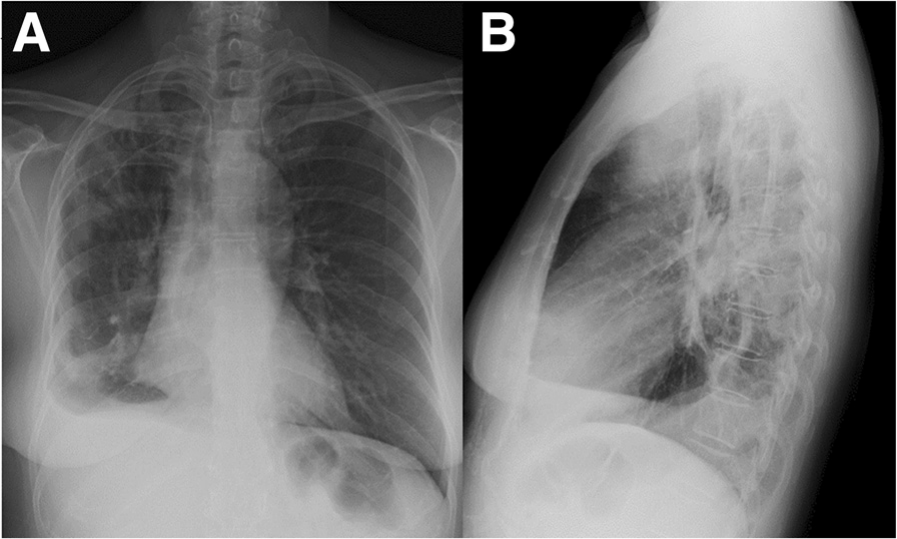
Chest X-ray showing peripheral pulmonary infiltrates predominantly in the right lung with pleural effusion **a** posterioranterior film, **b** lateral film

**Fig. 2:**
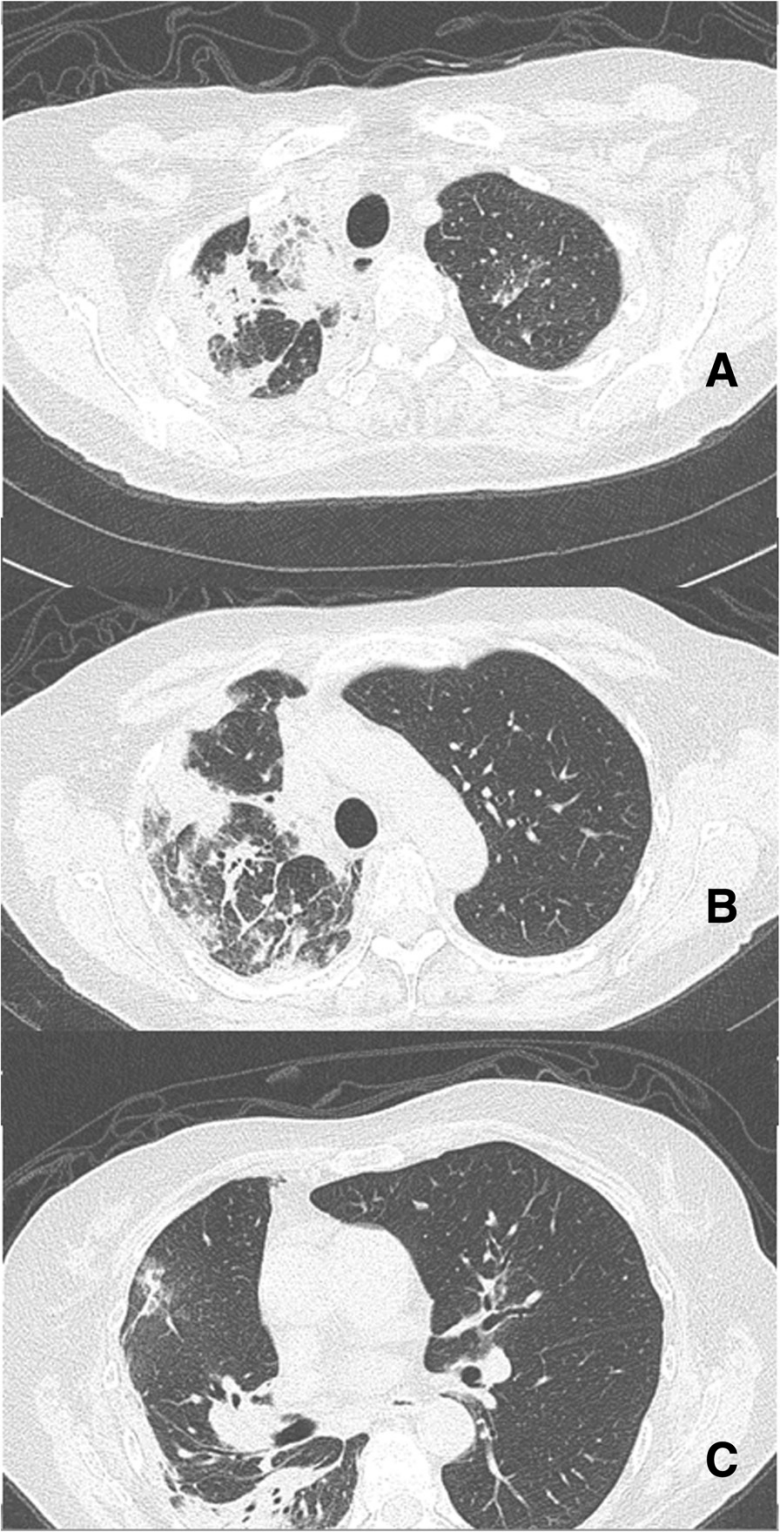
Chest computed tomography showing peripheral pulmonary opacities predominantly in the right lung **a** apical cut, **b** mid cut, **c** lower cut

**Fig. 3:**
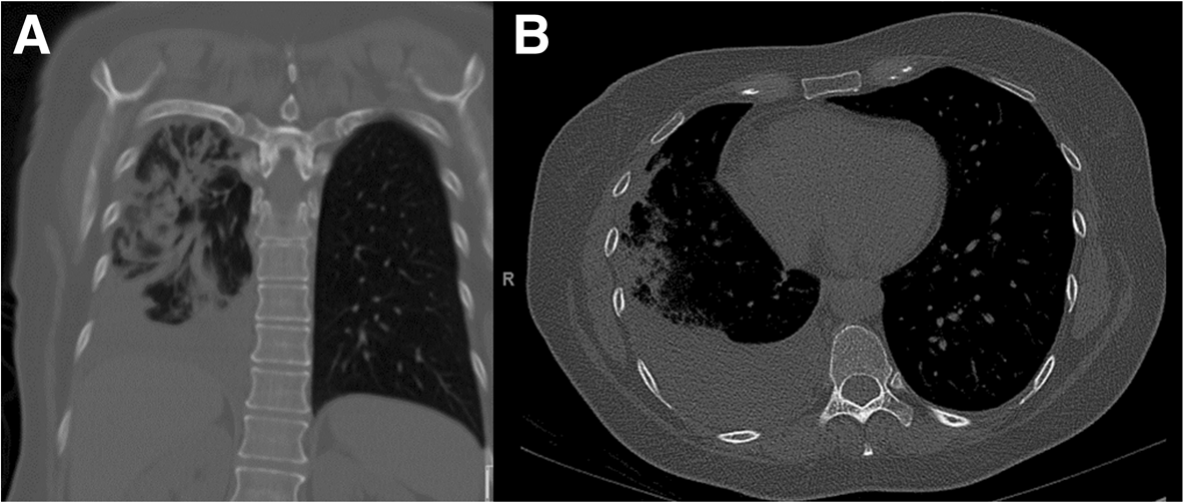
Chest computed tomography showing moderate-sized pleural effusion in the right chest ("**a**" and "**b**")

Laboratory studies revealed leukocytosis with a white blood cell count of 20.2 × 10^9^/L with a markedly elevated 16 % eosinophils (3.3 × 10^9^/L), 66 % neutrophils, and 12 % lymphocytes. Her hemoglobin level was 10.9 g/dL and platelet count was 392 × 10^9^/L. Her blood biochemical profiles as well as serum immunoglobulins were all unremarkable. Infectious disease etiologies workup including serologies for human immunodeficiency virus (HIV), coccidioides, blastomyces, cryptococcus, strongyloides, and toxocara were all negative. Her blood cultures, urine culture and sputum culture yielded no growth. Stool examinations for ova and parasites were negative. Vasculititides and connective tissue diseases workup including anti-nuclear antibody, anti-myeloperoxidase, anti-serine protease, anti-double-stranded DNA, rheumatoid factor, and cyclic citrullinated peptide were unremarkable. A chest X-ray showed multifocal consolidation predominantly in her right lung with a moderate-sized pleural effusion (Fig. 1). A chest computed tomography scan confirmed the findings seen on chest X-ray (Fig. 2 and 3). Echocardiographic findings were normal.

**Fig. 4:**
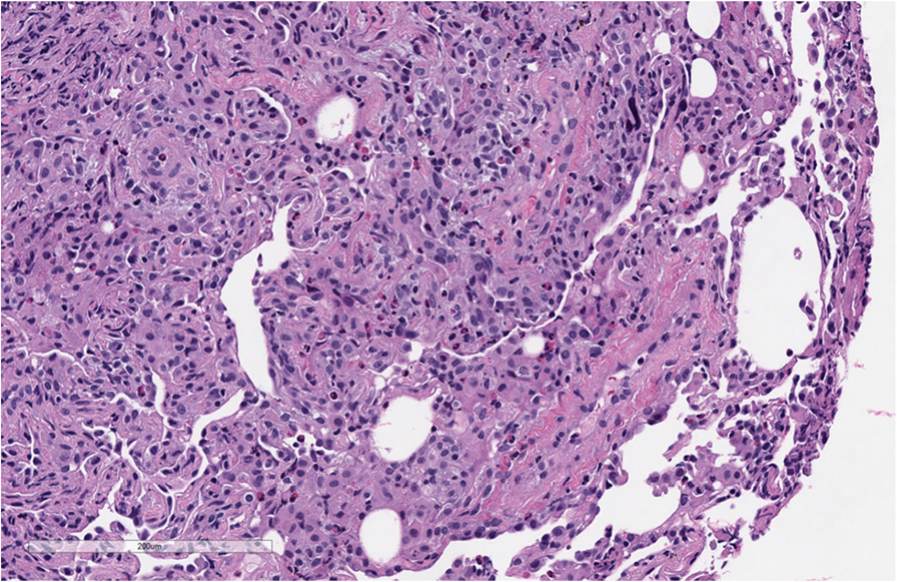
Transbronchial biopsy showing eosinophilic infiltrates within reactive lung parenchyma. No vasculitis or microorganisms were seen, H&E, ×200 magnification

**Fig. 5:**
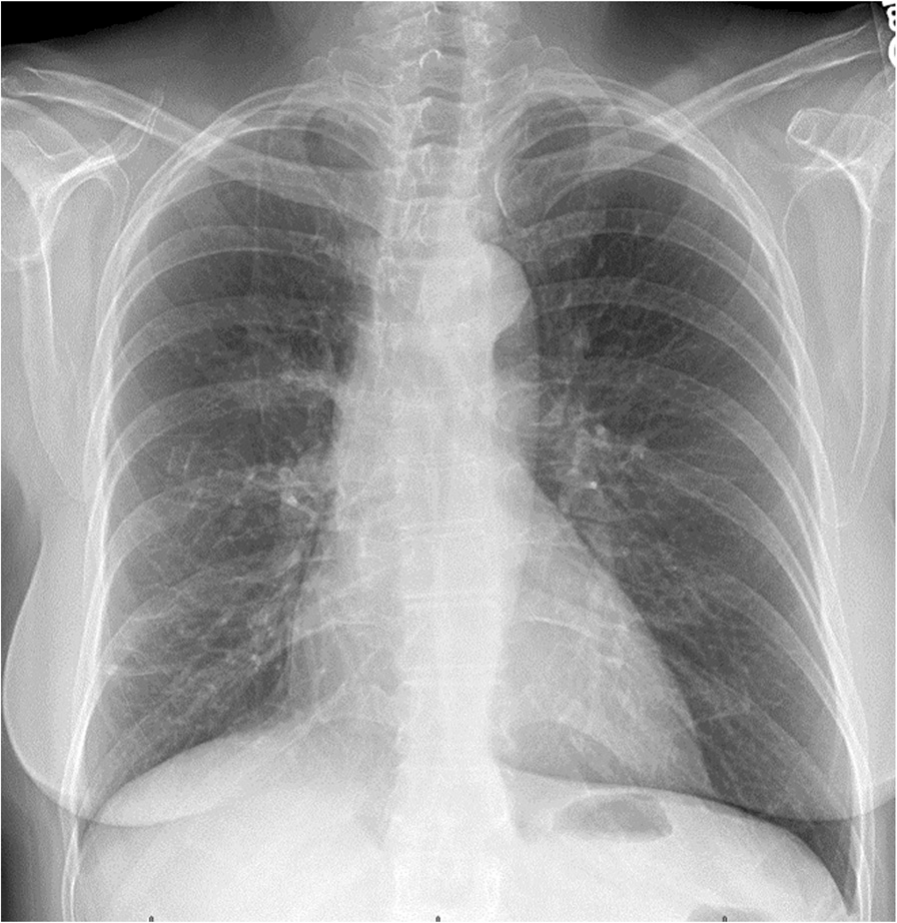
Chest X-ray showing resolution of pulmonary infiltrates and pleural effusion 1 month following steroid treatment

Our patient did not improve despite empirical antibiotic treatment with vancomycin and cefepime. She underwent thoracentesis of her right chest and bronchoscopy. Pleural fluid demonstrated a white blood cell count of 41,000 cells per mL with 38 % eosinophils, 32 % lymphocytes and 27 % neutrophils. Pleural fluid chemistries were consistent with a transudative profile with lactate dehydrogenous (LDH) of 70 U/L (serum LDH 184 U/L), protein 2.9 g/dL (serum protein 7 g/dL) and glucose 86 mg/dL. Bronchoalveolar lavage (BAL) fluid demonstrated a white blood cell count of 1985 cells per mL with 74 % eosinophils, 13 % lymphocytes and 7 % neutrophils. A transbronchial biopsy performed from the right lower lobe was suggestive of eosinophilic pneumonia with marked increased eosinophils without evidence of vasculitis, malignancy or fungal organisms (Fig. 4). The diagnosis of chronic eosinophilic pneumonia was made based on peripheral eosinophilia, eosinophilic pleural effusion, high percentage of BAL eosinophils, the finding of eosinophilic pneumonia on transbronchial biopsy, and the absence of other causes of eosinophilia. Our patient was started on steroids (prednisone 1 mg/kg/day at 60 mg/day) and all antibiotics were discontinued given no convincing evidence of infection. She had an excellent rapid clinical improvement. She returned to work 2 weeks after hospital discharge. A chest X-ray (Fig. 5) obtained 1 month after steroid treatment showed complete resolution of the consolidations and pleural effusion. Peripheral eosinophilia was resolved. Steroids were tapered gradually over the course of 1 year. Our patient relapsed when the prednisone dose was decreased to 2.5 mg/day. She responded again when the prednisone dose was increased to 20 mg/day. She slowly tapered steroids over another year and she had been off of steroids for 2 months at the time of report. There was no adverse side effect from steroids over the course of treatment, except for 4 kg of weight gain.

## Discussion

CEP is a rare disorder characterized by pulmonary infiltrates with eosinophils. There is no consensus on the diagnostic criteria. The diagnosis is typically made based on clinical presentation, chest radiographic findings of predominantly peripheral opacities or “photographic negative” of pulmonary edema and identification of eosinophilic infiltrates in the lungs either on biopsy or BAL samples [2, 4]. Most patients with CEP have peripheral blood eosinophilia, which is defined as greater than 6 % of the total white blood cell count [5]. The mainstay of treatment is corticosteroids and most patients required a relatively extended course of therapy [6]. Thus, it is crucial that the diagnosis of CEP should only be made after the exclusion of other eosinophilic lung diseases such as parasitic and fungal infections, medication-induced eosinophilic pneumonia, malignancy (both solid and hematologic malignancies), hypereosinophilic syndrome, and vasculitides. The clinical presentation, typical radiographic findings and evidence of eosinophils in pulmonary infiltrates in the BAL, pleural fluid, and transbronchial biopsy specimens in our patient suggest the diagnosis of CEP. The excellent response to corticosteroid treatment is another characteristic feature of CEP [6] and helped confirming the diagnosis in this patient. If the patient did not improve and peripheral eosinophilia still persists, we would have planed to perform a bone marrow biopsy and obtain FIP1L1-PDGFR alpha to evaluate bone marrow diseases such as myeloproliferative neoplasms and hypereosinophilic syndrome.

Our case illustrated two unique radiographic manifestations of CEP. First, the patient had ipsilateral pleural effusion, an uncommon radiographic finding in CEP. In two studies, only four of 81 patients had pleural effusions [5, 6]. We identified six other reported cases of CEP with effusions [7–12]. In addition, the pleural fluid chemistries in our case were consistent with a transudative effusion, in contrast to exudative effusions reported in the three other cases of CEP with pleural effusion [10–12]. The mechanism of pleural effusion development in CEP is unclear. The transudative profile suggests an increase in microvascular permeability in our case. In all cases of CEP with pleural effusion including our case, effusion resolved with corticosteroids treatment. Second, this patient demonstrated nonsegmental peripheral airspace consolidation (photographic negative of pulmonary edema), a frequent finding in chest radiographs of CEP. However, the distribution in this patient was unilateral, predominantly involving her right lung, while most CEP cases have bilateral lung involvement [4]. In one case series, unilateral infiltrates on chest X-ray occurred in six of 12 patients [13].

In this case, diagnostic thoracentesis was performed prior to bronchoscopy. The finding of pleural fluid eosinophilia can be nonspecific but it was helpful to rule out parapneumonic effusion and empyema. We proceeded with diagnostic bronchoscopy with transbronchial biopsies to rule out infectious etiologies. This also helped us to confirm the diagnosis of CEP prior to initiation of a prolonged course of steroid treatment. In our opinion, the presence of pleural fluid eosinophilia is a rare presentation of CEP and it should not be used to make the diagnosis of CEP. As in other CEP cases without pleural effusion, diagnosis of CEP should only be made carefully in the right clinical context and exclusion of other eosinophilic lung diseases. Once diagnosis is made, the treatment of CEP with effusion is similar to CEP without effusion.

## Conclusions

This is a rare case of CEP presenting with transudative eosinophilic pleural effusion. Similar to other cases of CEP, early recognition, diagnosis, and prompt treatment with corticosteroids are the mainstay of therapy. In this case pleural effusion resolved without the need for therapeutic thoracentesis.

## Abbreviations

ADL, activities of daily living; BAL, bronchoalveolar lavage; CEP, chronic eosinophilic pneumonia; DNA, deoxyribonucleic acid; H&E, hematoxylin and eosin; HIV, human immunodeficiency virus; LDH, lactate dehydrogenous

## References

[1] Thomeer MJ, Costabe U, Rizzato G, Poletti V, Demedts M. Comparison of registries of interstitial lung diseases in three European countries. Eur Respir J Suppl. 2001;32:114s–8s. 11816817

[2] Marchand E, Cordier JF. Idiopathic chronic eosinophilic pneumonia. Orphanet J Rare Dis. 2006;1:11. 10.1186/1750-1172-1-11 PMC1464381 16722612

[3] Jeong YJ, Kim KI, Seo IJ, Lee CH, Lee KN, Kim KN, Kim JS, Kwon WJ. Eosinophilic lung diseases: a clinical, radiologic, and pathologic overview. Radiographics. 2007;27:617–37. discussion 637-619. 10.1148/rg.273065051 17495282

[4] Allen JN, Davis WB. Eosinophilic lung diseases. Am J Respir Crit Care Med. 1994;150:1423–38. 10.1164/ajrccm.150.5.7952571 7952571

[5] Jederlinic PJ, Sicilian L, Gaensler EA. Chronic eosinophilic pneumonia. A report of 19 cases and a review of the literature. Medicine (Baltimore). 1988;67:154–62. 10.1097/00005792-198805000-00002 3285120

[6] Marchand E, Reynaud-Gaubert M, Lauque D, Durieu J, Tonnel AB, Cordier JF. Idiopathic chronic eosinophilic pneumonia. A clinical and follow-up study of 62 cases. The Groupe d’Etudes et de Recherche sur les Maladies “Orphelines” Pulmonaires (GERM“O”P). Medicine (Baltimore). 1998;77:299–312. 10.1097/00005792-199809000-00001 9772920

[7] Rutgers SR, Schweitzer M. Chronic eosinophilic pneumonia with pleural effusion. Neth J Med. 1999;54:191–6. 10.1016/s0300-2977(99)00028-5 10365560

[8] Puchala M, Samel S, Schwartzova I, Matejny B, Rovensky E, Funk M, Komada J. Eosinophilic pleural exudate in chronic eosinophilic pneumonia (Carrington’s pneumonia). Vnitr Lek. 1981;27:889–94. 7293033

[9] Harrow EM. Eosinophilic pleural effusion in a patient with chronic eosinophilic pneumonia. J Maine Med Assoc. 1980;71:241–3. 7240950

[10] d’Amours P, Leblanc P, Boulet LP. Chronic eosinophilic pneumonia associated with thrombocytosis and pleural effusion. CMAJ. 1990;142:837–9. PMC1451919 2322915

[11] Jee YG, Ra SH, Park YM, Cha JW, Kang YS, Park JH, Kang TY. A case of rheumatoid arthritis with chronic eosinophilic pneumonia associated with eosinophilic pleural effusion. J Rheum Dis. 2013;20:328–31.

[12] Samman YS, Wali SO, Abdelaal MA, Gangi MT, Krayem AB. Chronic eosinophilic pneumonia presenting with recurrent massive bilateral pleural effusion: case report. Chest. 2001;119:968–70. 10.1378/chest.119.3.968 11243986

[13] Naughton M, Fahy J, FitzGerald MX. Chronic eosinophilic pneumonia. A long-term follow-up of 12 patients. Chest. 1993;103:162–5. 10.1378/chest.103.1.162 8031327

